# Creatine and taurine mixtures alleviate depressive-like behaviour in *Drosophila melanogaster* and mice via regulating Akt and ERK/BDNF pathways

**DOI:** 10.1038/s41598-020-68424-1

**Published:** 2020-07-09

**Authors:** Suhyeon Kim, Ki-Bae Hong, Singeun Kim, Hyung Joo Suh, Kyungae Jo

**Affiliations:** 1 Department of Integrated Biomedical and Life Science, Graduate School, Korea University, Seoul, 02841 Republic of Korea; 2 BK21Plus, College of Health Science, Korea University, Seoul, 02841 Republic of Korea

**Keywords:** Chemical biology, Immunology, Molecular biology, Neuroscience

## Abstract

We investigated the antidepressant effect of creatine (CRE) and taurine (TAU) mixtures on behavioural changes and biomarkers in stress-induced depression in *Drosophila melanogaster* and a mouse model. Following CRE/TAU mixture administration in the *Drosophila* model, depression-like state induced by vibration, locomotion, climbing activity, and survival rate were measured. The normal stress (NS) group demonstrated decreased movement than the control (CON) group; movements in the CRE/TAU-treated group (particularly 0.15/0.5%) returned to the CON levels. Antidepressant effects of CRE/TAU mixtures were confirmed in a depressive mouse model induced by chronic mild stress. In behavioural assessments, movement and sucrose preference of the CRE/TAU group increased to a similar level as in the positive control group; hippocampal catecholamine and serotonin levels increased significantly. Stress-related hormones (adrenocorticotropic and corticotropin-releasing hormones) and inflammatory factors (IL-1β, IL-6, and TNF-α) increased in the NS group but significantly decreased in the CRE/TAU-treated group. Brain signalling protein expression ratio of phosphorylated protein kinase B (p-Akt)/Akt, phosphorylated extracellular signal-regulated kinase (p-ERK)/ERK, and brain-derived neurotrophic factor (BDNF) significantly increased in the CRE/TAU-treated group. These results indicate that CRE/TAU-induced antidepressant effects are associated with increased behavioural patterns and downregulation of stress hormones and cytokines, mediated through Akt and ERK/BDNF pathways in vertebrate models.

## Introduction

Depression, commonly known to render a person’s mental state or physical activity incapacitated, is classified as one of the most dangerous diseases in modern society according to the World Health Organization (WHO), with a prevalence of 15–25% of the world’s population^1,2^. The etiology of depression remains unclear; however, biological, genetic, and social psychological factors are thought to interact with each other^3,4^.

Currently, most patients with depression are treated with synthetic drugs, including selective serotonin reuptake inhibitors (SSRI), serotonin-norepinephrine reuptake inhibitors, tricyclic antidepressants, norepinephrine and dopamine reuptake blockers, and monoamine oxidase inhibitors^5^. However, these chemically synthesised antidepressants have low reactivity and recovery rates and have side effects such as fatigue, sleep disorders, cognitive disorders, and sexual dysfunctions^6,7^. Therefore, several studies have recently been undertaken owing to the increased demand for naturally derived antidepressants from medicinal plants and food-containing ingredients.

The important physiological role of creatine (CRE) in depression has been confirmed in several clinical studies^8–10^. Reportedly, there is a decrease in phosphocreatine in the brains of severely depressed patients^8^. In particular, CRE supplements improve the mood of individuals with post-traumatic stress disorder, depression, fibromyalgia^9^, and therapy-resistant depression^10^. However, owing to concerns regarding the theoretical side effects, including kidney, muscle, and thermal dysfunctions and cramps and gastrointestinal discomfort, associated with CRE supplements^11^, we selected a mixture with the lowest amount of CRE. Additionally, taurine (TAU), 2-aminoethanesulfonic acid (C_2_H_7_NO_3_S), is one of the most abundant free amino acids in the central nervous system, including hypothalamic neuroglial cells^12^, and exhibits anxiolytic-like effects in mice and rats by acting as an inhibitory neurotransmitter through gamma-aminobutyric acid (GABA) and glycine receptors^13,14^. The increase in TAU in these depression models may play an effective role in the brain.

In this study, we investigated the activity of CRE and TAU, which have antidepressant effects, and confirmed the mechanism of action. First, the optimal combination ratio of CRE and TAU, which are more synergistic than when used alone, was selected through a *Drosophila melanogaster* experiment with vibration stress. Then, BALB/c mice with induced chronic mild stress (CMS) were treated with CRE, TAU, and CRE/TAU mixture, and their antidepressant effect and mechanism were evaluated by behavioural and biomarker analyses. The findings would provide insights on the effectiveness of CRE/TAU mixtures in ameliorating depression.

## Results

### Effects of CRE and TAU on locomotor activity of vibration-stressed *D. melanogaster*

**Figure 1:**
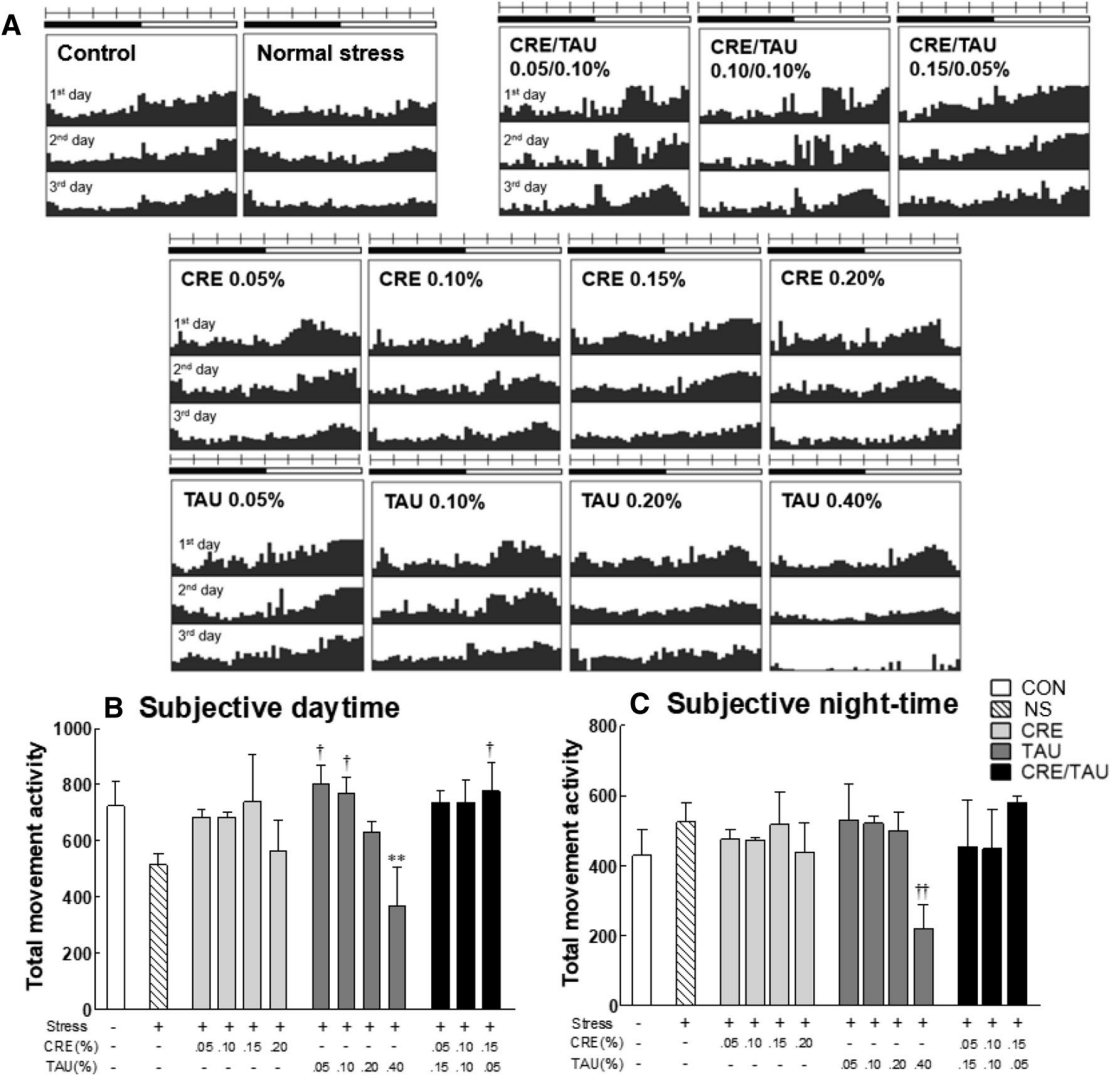
Effects of creatine (CRE), taurine (TAU), and the mixture of CRE and TAU on locomotor activity of vibration-stressed *Drosophila melanogaster*. (**A**) Representative actograms for the locomotor activity of non-stressed flies (control) (n = 50) and flies exposed to vibration stress (normal stress) (n = 50) and CRE, TAU, or ratio of CRE/TAU mixtures (n = 50, respectively). Activity was measured as counts per min, and then all activities for 30 min were combined and calculated over 3 d. The bar above each actogram indicates day (in white) and night (in black) h. (**B**) Activity during subjective daytime and (**C**) night-time. Results are presented as the mean ± standard error of the mean (SEM) for each group. **p < 0.01 versus control group; ^†^*p* < 0.05 and ^**††**^*p* < 0.01 versus normal stress group (ANOVA followed by post-hoc Tukey’s test).

Actograms demonstrated that the CRE/TAU mixture effectively regulated the circadian rhythm of *Drosophila* under stress conditions, as compared to both time zones (day and night) of the control (CON) group a (Fig. 1A). Compared to the CON group, the normal stress (NS) group showed decreased daytime activity (Fig. 1B; F(12,637) = 6.237, η^2^_p_ = 0.742) and increased night-time activity (Fig. 1C; F(12,637) = 3.909, η^2^_p_ = 0.643). The TAU (0.05, 0.10%) groups showed a significant increase in daytime activity when compared to that in the NS group (*p* < 0.016 and *p* < 0.046, respectively); however, high concentration of TAU (0.40%) tended to significantly decrease both day- (vs. CON group, *p* < 0.002) and night-time activity (vs. NS group, *p* < 0.003). Therefore, the maximum concentration of CRE and TAU was set as 0.20%, and the mixed media in the ratio of 1:3, 1:1, and 3:1 were provided to *Drosophila*. In groups treated with CRE and TAU, in combination with stress, the day- and night-time activities were restored to levels more comparable with those in the CON group than in the CRE and TAU alone groups.

### Effects of CRE and TAU on behavioural pattern in vibration-stressed *D. melanogaster*

**Figure 2:**
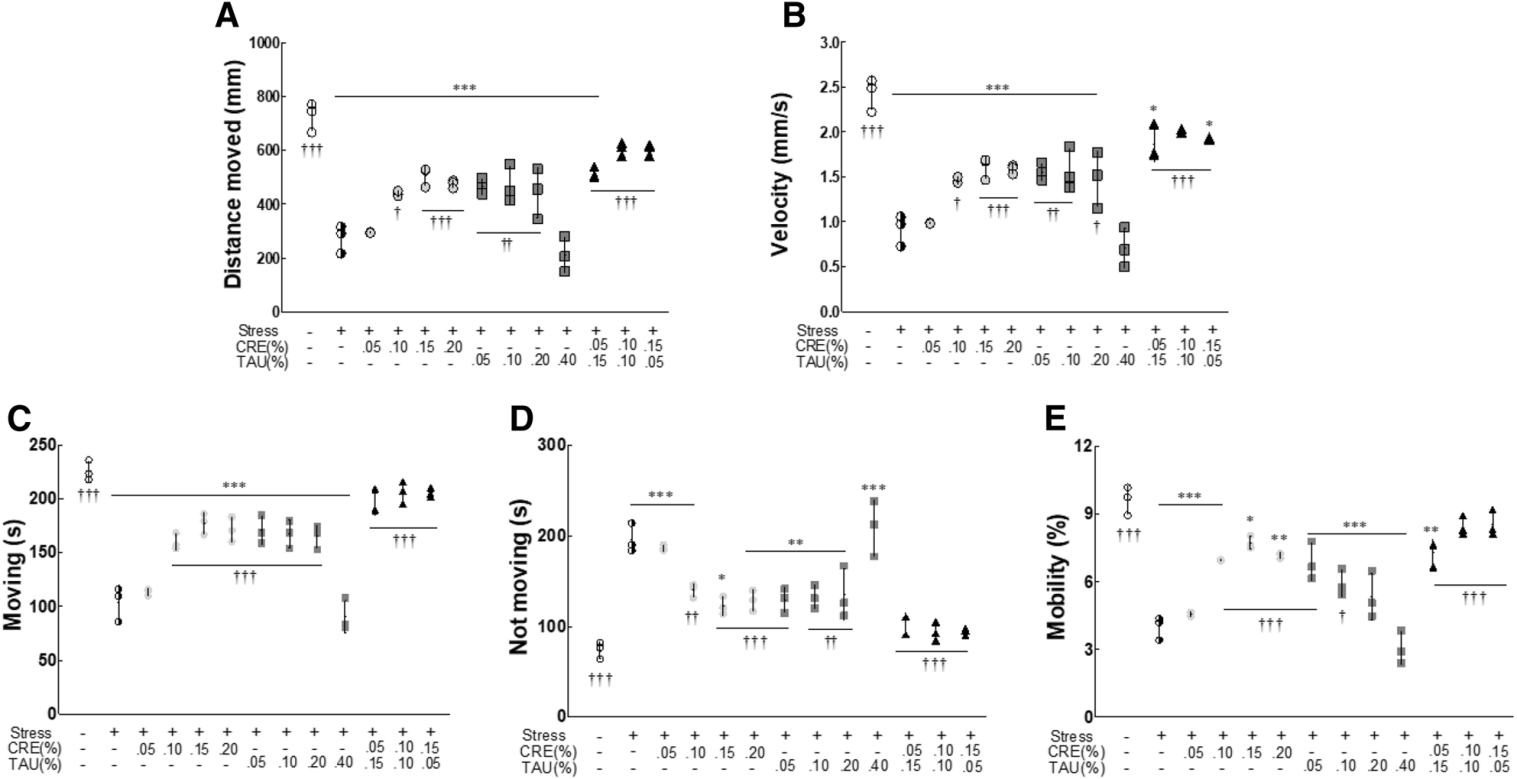
Effects of creatine (CRE), taurine (TAU), and the mixture of CRE and TAU on behavioural patterns of vibration-stressed *Drosophila melanogaster*. Experimental flies were analysed using the Noldus EthoVision-XT system in the non-stressed group, stressed group, CRE-treated groups (0.05, 0.10, 0.15, and 0.20%), TAU-treated groups (0.05, 0.10, 0.20, and 0.40%), and CRE/TAU mixture-treated groups (0.05/0.15, 0.10/0.10, and 0.15/0.05%) with vibration stress. *p < 0.05, **p < 0.01, and ***p < 0.001 versus control group; ^†^*p* < 0.05, ^††^*p* < 0.01, and ^†††^*p* < 0.001 versus normal stress group (ANOVA followed by post-hoc Tukey’s test).

The analysis indexes of video tracking were the distance moved (Fig. 2A; F(12,637) = 28.369, η^2^_p_ = 0.909), velocity (Fig. 2B; F(12,637) = 26.261, η^2^_p_ = 0.924), moving (Fig. 2C; F(12,637) = 41.703, η^2^_p_ = 0.951), not moving (Fig. 2D; F(12,637) = 21.890, η^2^_p_ = 0.910), and mobility (Fig. 2E; F(12,637) = 34.016, η^2^_p_ = 0.940). The NS group demonstrated a significant decrease in all indexes compared to that in the CON group, except for the index not moving (Fig. 2, *p* < 0.001). CRE groups (0.05%, 0.10%, and 0.15%) demonstrated a concentration-dependent increase in distance moved, velocity, moving, and mobility; notably, CRE 0.15% and CRE 0.20% groups exhibited a significant increase in the aforementioned indexes when compared to that in the NS group (*p* < 0.05). The TAU group demonstrated an increase in distance moved, velocity, moving, and mobility at low concentrations (from 0.05% to 0.20%) and a decrease at high concentration (0.40%). In contrast, in the CRE/TAU mixture groups (0.05/0.15%, 0.1/0.1%, and 0.15/0.05%), the distance moved, velocity, moving, and mobility significantly increased when compared to that in the NS group (*p* < 0.001). Therefore, the CRE/TAU mixtures induced behavioural changes and *Drosophila* were more active than when treated with CRE or TAU alone, indicating recovery to similar levels as that in the CON group.

### Effect of CRE and TAU on climbing activity of vibration-stressed *D. melanogaster*

**Figure 3:**
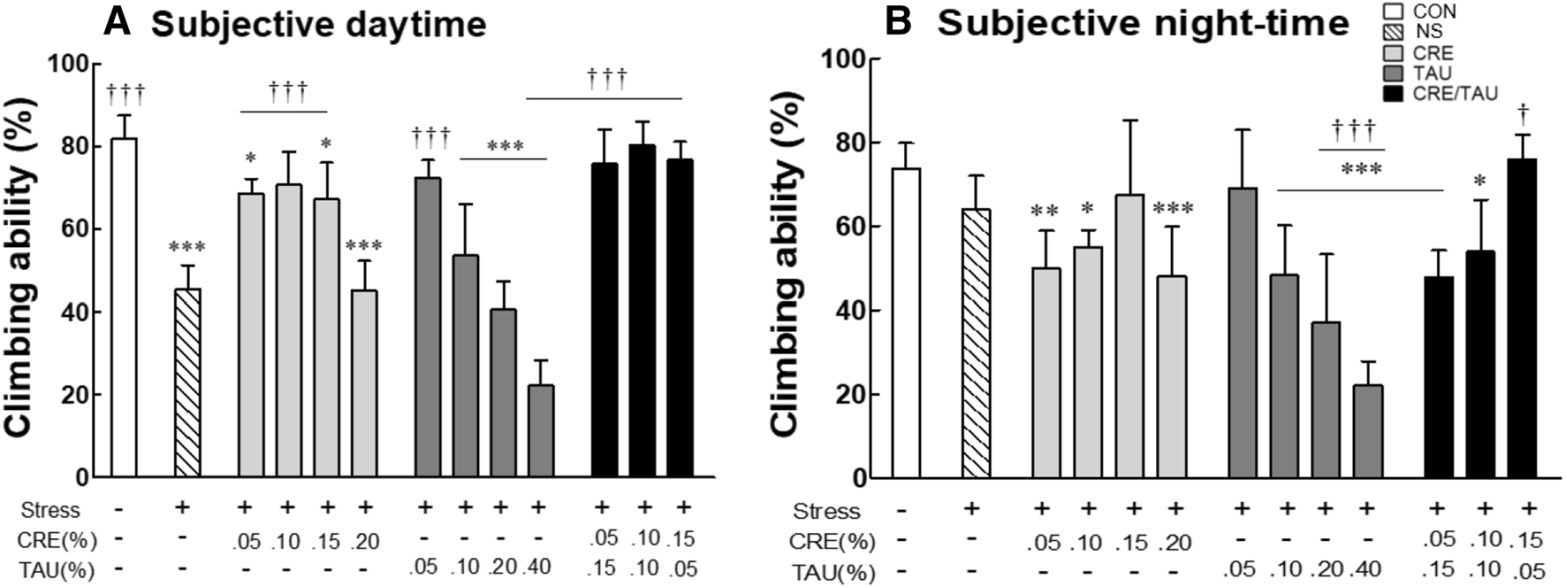
Effects of creatine (CRE), taurine (TAU), and the mixture of CRE and TAU on climbing activity of vibration-stressed *Drosophila melanogaster*. Experiments analysed the climbing ability during the (**A**) subjective daytime and (**B**) night-time in the non-stressed group, stressed group, CRE-treated groups (0.05, 0.10, 0.15, and 0.20%), TAU-treated groups (0.05, 0.10, 0.20, and 0.40%), and the CRE/TAU mixture-treated groups (0.05/0.15, 0.10/0.10, and 0.15/0.05%) with vibration stress. Results are presented as the mean ± standard error of the mean (SEM) for each group. *p < 0.05, **p < 0.01, and ***p < 0.001 versus control group; ^†^*p* < 0.05 and ^†††^*p* < 0.001 versus normal stress group (ANOVA followed by post-hoc Tukey’s test).

At the subjective daytime, the climbing ability of the NS group was significantly lower than that in the CON group (Fig. 3A; F(12,637) = 47.769, η^2^_p_ = 0.880, *p* < 0.001). Furthermore, the climbing abilities of the CRE groups (0.05%, 0.1%, and 0.15%), TAU group (0.05%), and CRE/TAU mixture groups (0.15/0.05%, 0.10/0.10%, 0.05/0.15%) significantly increased when compared with that in the NS group (*p* < 0.001). In contrast, during the subjective night-time, the climbing ability of the NS group decreased when compared with that in the CON group, but no significant difference was observed (Fig. 3B; F(12,637) = 16.105, η^2^_p_ = 0.680, p = 0.833). The subjective night-time climbing ability increased in the CRE 0.15% and TAU 0.05% groups compared to that in the NS group, but there was no significant difference. However, a significant increase was observed in the CRE/TAU mixture (0.15/0.05%) group (p = 0.046). In conclusion, the use of CRE/TAU mixture resulted in a more effective antidepressant activity than that by the use of CRE and TAU alone in stress-induced depression *Drosophila* model. In the case of TAU, the antidepressant activity was low in the high concentration treated group, and especially in the TAU 0.15% group, the survival rate decreased by 39% after 6 days of treatment (Supplementary Fig. 1). Therefore, we selected the 3:1 ratio (CRE/TAU 0.15/0.05%) with a low TAU concentration and high antidepressant activity among the CRE/TAU mixture groups and then confirmed the antidepressant effect in the mouse model.

### Effect of CRE and TAU on body and organ weight in chronic mild stressed mice

The body weight of the CON group increased during the entire experimental period, whereas the NS group demonstrated a significant decrease when compared to that of the CON group after 5 weeks of CMS (Supplementary Table 2; F(5,42) = 8.984, η^2^_p_ = 0.517, *p* < 0.001). Fluoxetine (10 mg/kg)-administered (FLU) group, as the positive control, demonstrated results similar to the CON group and the body weight increased during the duration of the experiment. At week 5, the CRE and TAU groups significantly gained weight compared to that of the NS group (*p* < 0.024 and *p* < 0.049, respectively). Conversely, the CRE/TAU group demonstrated body weight gain similar to that in the FLU group, and the body weight significantly increased when compared to that of the NS group (*p* < 0.002).

After 5 weeks of experimentation, the animals were euthanized, and the kidney, spleen, liver, heart, total brain, and hippocampus weights were measured immediately (Supplementary Table 3). The kidney, spleen, heart, and hippocampus weights did not significantly differ between groups. On the contrary, compared to the CON group, in the NS group, the liver weights significantly decreased (F(5,42) = 14.459, η^2^_p_ = 0.633, *p* < 0.002), and the FLU, CRE, TAU, and CRE/TAU groups demonstrated an increased weight similar to that in the CON group (*p* < 0.001).

### Effect of CRE and TAU on sucrose preference in CMS-induced depressive mice

**Figure 4:**
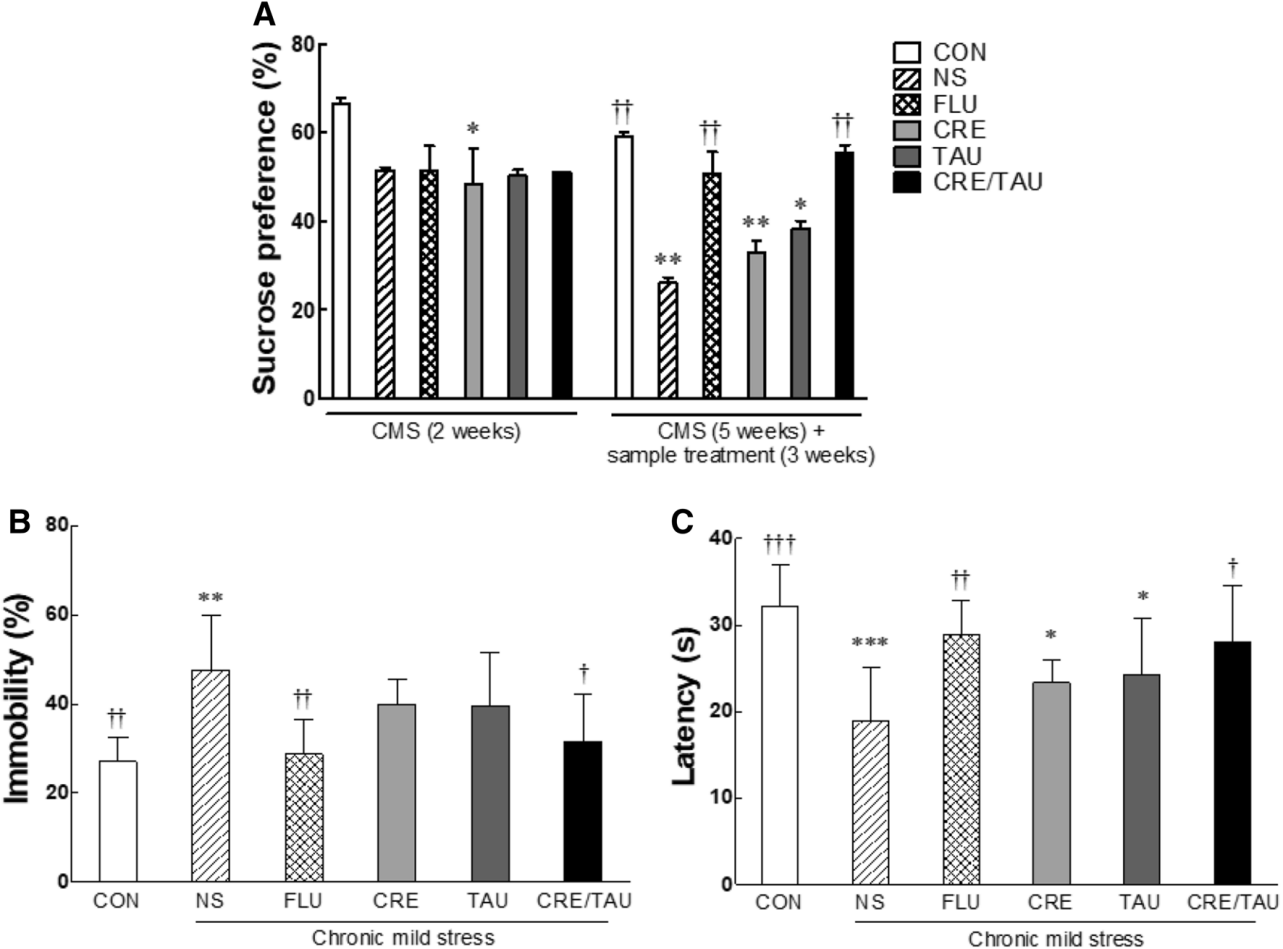
Effects of creatine, taurine, and the mixture of creatine and taurine on behaviours of chronic mild stressed mice. After five weeks of chronic mild stress treatment, all groups of mice underwent behavioural evaluation tests, including the (**A**) sucrose preference test and (**B**,**C**) forced swimming test. CON: non-stressed group (control), NS: stressed group (normal stress), FLU: 10 mg/kg of fluoxetine treatment (positive control), CRE: 7.5 mg/kg of creatine, TAU: 2.5 mg/kg of taurine, CRE/TAU: 7.5 mg/kg of creatine and 2.5 mg/kg of taurine mixture. Results are presented as the mean ± standard error of the mean (SEM) for each group (n = 8). *p < 0.05 and **p < 0.01 versus control group. ^†^*p* < 0.05, ^††^*p* < 0.01, and ^†††^*p* < 0.001 versus normal stress group (ANOVA followed by post-hoc Tukey’s test).

The sucrose preference test was first conducted after subjecting the mice to CMS and then after oral administration of the samples with the CMS. In the first test, the sucrose preferences of all stressed groups, except the CON group, tended to decrease (Fig. 4A; F(5,42) = 5.151, η^2^_p_ = 0.811). In the second test, the sucrose preference of the NS group significantly decreased when compared to that of the CON group (F(5,42) = 24.658, η^2^_p_ = 0.954, *p* < 0.001). In contrast, compared with the NS group, in the FLU and CRE/TAU groups, sucrose preference significantly increased (*p* < 0.001 and *p* < 0.002, respectively) and was similar to that of the CON group.

### Effect of CRE and TAU on the forced swim test (FST) in CMS-induced depressive mice

The FST was performed for 5 min, and immobility and the latency to the first episode of immobility were measured. Immobility increased significantly in the NS group (Fig. 4B; F(5,42) = 5.650, η^2^_p_ = 0.042, *p* < 0.001) compared with that in the CON group. FLU and CRE/TAU groups demonstrated significantly lower immobility than the NS group (*p* < 0.003 and *p* < 0.018, respectively). CRE and TAU groups showed a decrease in immobility time when compared with that in the NS group, with no significant difference. Moreover, the latency in the NS group significantly decreased when compared with that in the CON group (Fig. 4C; F(5,42) = 6.327, η^2^_p_ = 0.043, *p* < 0.001). Contrarily, the latency increased in all groups when compared with that in the NS group. Notably, the latency in the FLU and CRE/TAU groups increased significantly when compared with that in the NS group (*p* < 0.007 and *p* < 0.016, respectively).

### Effect of CRE and TAU on the open field test (OFT) in CMS-induced depressive mice

**Figure 5:**
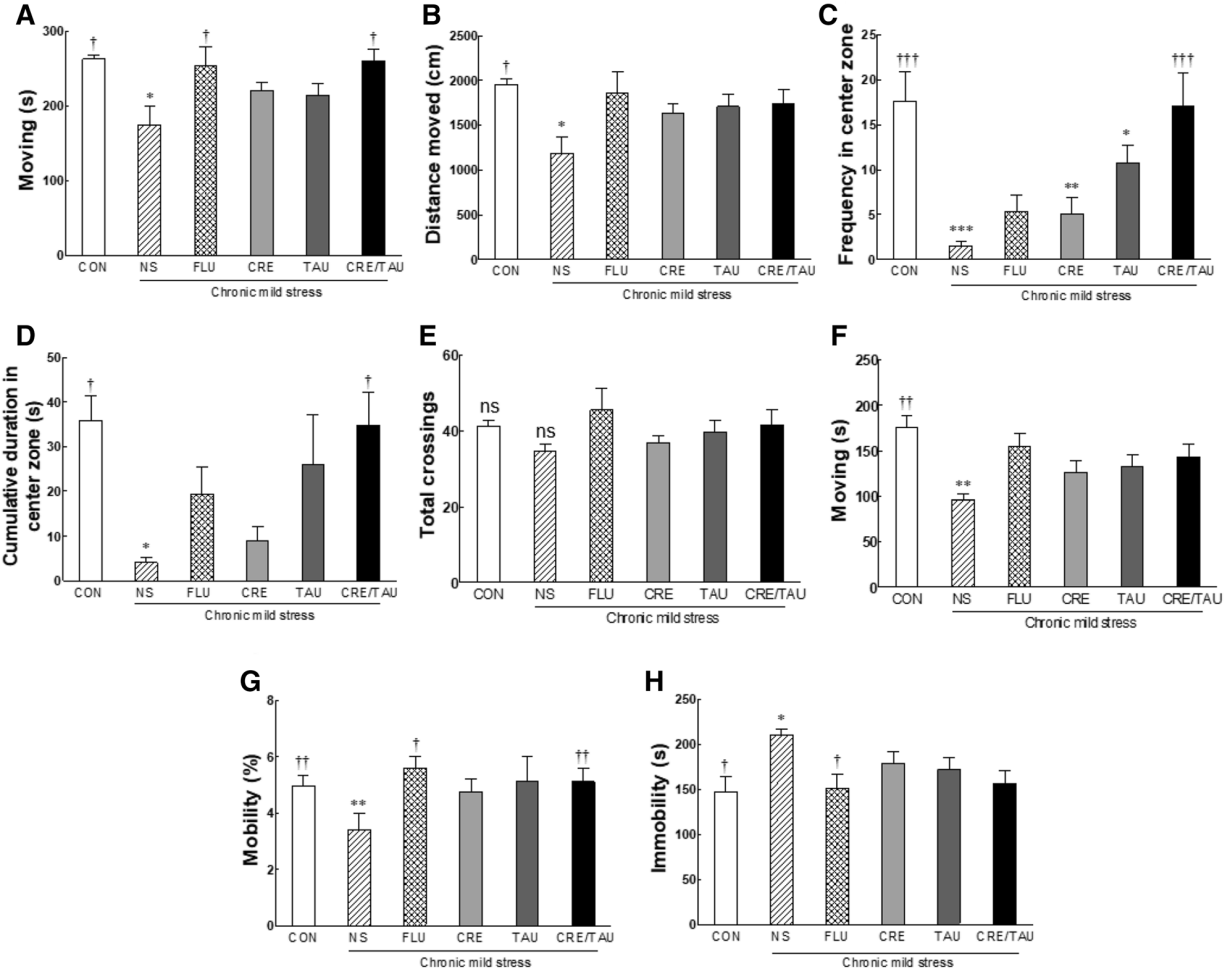
Effects of creatine, taurine, and the mixture of creatine and taurine on locomotor activity of chronic mild stressed mice. After five weeks of chronic mild stress treatment, all mice groups underwent behavioural evaluation tests, including the (**A**–**E**) open field test and (**F**–**H**) tail suspension test. CON: non-stressed group (control), NS: stressed group (normal stress), FLU: 10 mg/kg of fluoxetine treatment (positive control), CRE: 7.5 mg/kg of creatine, TAU: 2.5 mg/kg of taurine, CRE/TAU: 7.5 mg/kg of creatine and 2.5 mg/kg of taurine mixture. Results are presented as the mean ± standard error of the mean (SEM) for each group (n = 8). *p < 0.05, **p < 0.01, and ***p < 0.001 versus control group; ^†^*p* < 0.05, ^††^*p* < 0.01, and ^†††^*p* < 0.001 versus normal stress group (ANOVA followed by post-hoc Tukey’s test).

Indicators identified in the OFT included moving time, distance moved, frequency in the centre zone, cumulative duration in the centre zone, and total crossing. In the case of moving time (Fig. 5A; F(5,42) = 3.612, η^2^_p_ = 0.301) and distance moved (Fig. 5B; F(5,42) = 2.797, η^2^_p_ = 0.250), the NS group demonstrated a significant decrease when compared to those in the CON group (*p* < 0.016 and *p* < 0.018, respectively). However, compared with the NS group, in the FLU and CRE/TAU groups, the moving times significantly increased (*p* < 0.016 and *p* < 0.020, respectively), and the distance moved increased but was not significantly different. Furthermore, for the frequency (Fig. 5C; F(5,42) = 7.460, η^2^_p_ = 0.470) and cumulative duration (Fig. 5D; F(5,42) = 3.721, η^2^_p_ = 0.312) in the centre zone, the NS group demonstrated a significant decrease when compared to those in the CON group (*p* < 0.001 and *p* < 0.030, respectively). The FLU, CRE, and TAU groups showed increased frequency and cumulative duration in the centre zone when compared to those in the NS group, but the difference was not significant. Moreover, compared with the NS group, the CRE/TAU group showed significantly increased frequency and cumulative duration in the centre zone (*p* < 0.001 and *p* < 0.035, respectively). Conversely, Fig. 5E shows that CMS, CRE, and TAU did not induce any significant changes compared with the CON group in horizontal ambulation in the open field (F(5,42) = 1.283, η^2^_p_ = 0.133).

### Effect of CRE and TAU on the tail suspension test (TST) in CMS-induced depressive mice

In the TST, moving time (Fig. 5F; F(5,42) = 4.365, η^2^_p_ = 0.342) and the mobility (Fig. 5G; F(5,42) = 5.142, η^2^_p_ = 0.380) of the NS group were significantly decreased when compared to the CON group (*p* < 0.002 and *p* < 0.003, respectively). In the FLU group, compared to the NS group, the mobility was significantly increased (*p* < 0.019). In the groups treated with CRE or TAU alone, the moving time and mobility were increased when compared to the NS group, with no significant difference. In contrast, compared to the NS group, the CRE/TAU group reported a significant increase in the mobility (*p* < 0.006). Immobility time was significantly increased in the NS group compared to that in the CON group (Fig. 5H; F(5,42) = 2.730, η^2^_p_ = 0.245, *p* < 0.026). In addition, compared to the NS group, only the FLU group showed a significant decrease in immobility time (*p* < 0.044).

### Effect of CRE and TAU on catecholamine and serotonin in CMS-induced depressive mice

High performance liquid chromatography (HPLC) was used to analyse the catecholamine (L-DOPA, dopamine, and epinephrine) and 5-hydroxytryptamine (5-HT) levels in the brain of experimental animals (Table 1). Epinephrine levels were below the limit of quantification in all groups and could not be measured. Therefore, the sum of L-DOPA (F(5,42) = 16.806, η^2^_p_ = 0.667) and 5-HT (F(5,42) = 10.875, η^2^_p_ = 0.565) was significantly lower in the NS group when compared to the CON group (*p* < 0.001). In all sample treated groups, the content was increased, but in the CRE/TAU group, it was significantly increased by approximately 1.26 times, when compared to the NS group (*p* < 0.001). Especially, dopamine and 5-HT levels were 2.47 ± 0.07 and 2.20 ± 0.05 ng/mg of tissue, respectively, which significantly increased in the CRE/TAU group when compared to those in the NS group (*p* < 0.001).

**Table 1:** Effects of creatine, taurine, and the mixture of creatine and taurine on the brain concentration of catecholamine and serotonin following chronic mild stress in mice.

Concentration (ng/mg of tissue)	Group					
	CON	NS	FLU	CRE	TAU	CRE/TAU
L-DOPA	3.58 ± 0.12^†††^	2.61 ± 0.13***	3.09 ± 0.05*^,†^	3.02 ± 0.09**	2.40 ± 0.10***	3.10 ± 0.11*^,†^
Dopamine	2.02 ± 0.05	1.70 ± 0.06	1.86 ± 0.02*	2.20 ± 0.10^†^	2.26 ± 0.08^††^	2.47 ± 0.07***^,†††^
5-HT	2.33 ± 0.06^†††^	1.87 ± 0.04***	2.38 ± 0.03*^,†^	1.99 ± 0.06***	2.09 ± 0.05*^,†^	2.20 ± 0.05^†††^
Epinephrine	< LOQ					
Total	7.93 ± 0.08^†††^	6.18 ± 0.08***	7.33 ± 0.03***	7.21 ± 0.08*^,††^	6.75 ± 0.08***	7.77 ± 0.08^†††^

CON: non-stressed group (control), NS: stressed group (normal stress), FLU: 10 mg/kg of fluoxetine treatment (positive control), CRE: 7.5 mg/kg of creatine, TAU: 2.5 mg/kg of taurine, CRE/TAU: 7.5 mg/kg of creatine and 2.5 mg/kg of taurine mixture, LOQ: limit of quantification. Results are presented as the mean ± standard error of the mean (SEM) for each group (n = 8). *p < 0.05, **p < 0.01, and ***p < 0.001 versus control group; ^†^*p* < 0.05, ^††^*p* < 0.01, and ^†††^*p* < 0.001 versus normal stress group (ANOVA followed by post-hoc Tukey’s test).

### Effect of CRE and TAU on stress hormones, corticotropin-releasing hormone (CRH) mRNA expression, and cytokines in CMS-induced depressive mice

**Figure 6:**
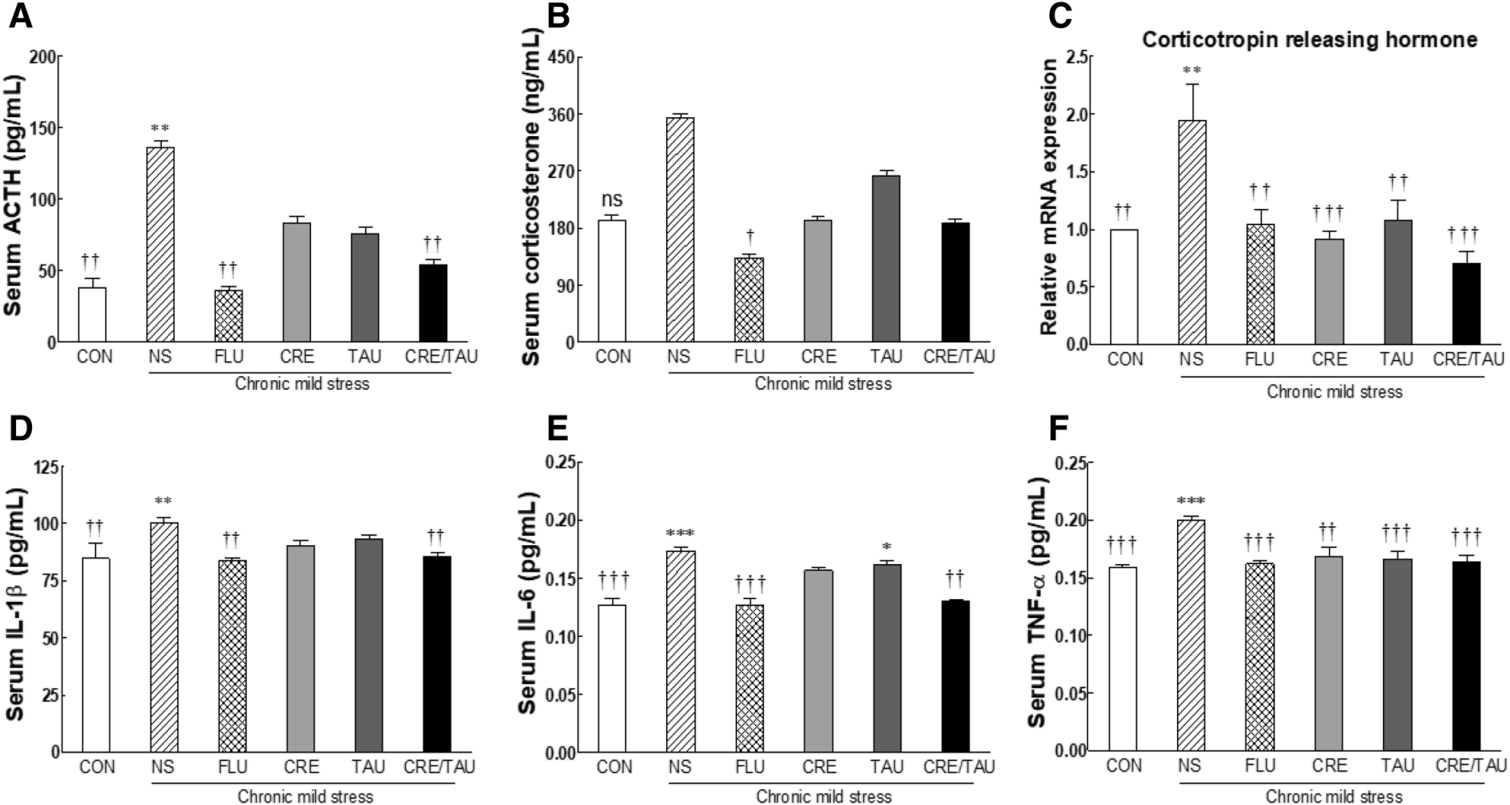
Effects of creatine, taurine, and the mixture of creatine and taurine on stress hormones, such as (**A**) adrenocorticotropic hormone (ACTH) and (**B**) corticosterone, (**C**) mRNA expression of corticotropin-releasing hormone (CRH) and cytokines, such as (**D**) IL-1β, (**E**) IL-6, and (**F**) TNF-α, in chronic mild stressed mice. CON: non-stressed group (control), NS: stressed group (normal stress), FLU: 10 mg/kg of fluoxetine treatment (positive control), CRE: 7.5 mg/kg of creatine, TAU: 2.5 mg/kg of taurine, CRE/TAU: 7.5 mg/kg of creatine and 2.5 mg/kg of taurine mixture. Results are presented as the mean ± standard error of the mean (SEM) for each group (n = 8). *p < 0.05, **p < 0.01, and ***p < 0.001 versus control group; ^†^*p* < 0.05, ^††^*p* < 0.01, and ^†††^*p* < 0.001 versus normal stress group (ANOVA followed by post-hoc Tukey’s test).

Levels of stress hormones, including adrenocorticotropic hormone (ACTH) and corticosterone, were measured by serum analysis. In the NS group, ACTH levels significantly increased when compared to that in the CON group (Fig. 6A; F(5,42) = 5.909, η^2^_p_ = 0.552, *p* < 0.005). Compared to the NS group, ACTH and corticosteroid levels (Fig. 6B; F(5,42) = 3.501, η^2^_p_ = 0.294) decreased in all groups; especially, ACTH levels in the FLU and CRE/TAU groups showed a significant decrease (*p* < 0.003), similar to the CON levels.

Therefore, we analysed the gene expression of the CRH, which stimulates the secretion of ACTH in the pituitary gland. As observed with the trend in the ACTH levels, the CRH mRNA expression levels in the NS group significantly increased when compared with those in the CON group (Fig. 6C; F(5,42) = 6.793, η^2^_p_ = 0.274, *p* < 0.002). The levels of CRH mRNA expression in the FLU (*p* < 0.003), CRE (*p* < 0.001), TAU (*p* < 0.005), and CRE/TAU groups (*p* < 0.001) significantly reduced compared to those in the NS group, similar to the levels in the CON group. In particular, the CRE/TAU group demonstrated a 2.75-fold decrease in the CRH mRNA expression levels when compared with that in the NS group (*p* < 0.001).

The inflammatory factors in the serum, IL-1β (Fig. 6D; F(5,42) = 5.821, η^2^_p_ = 0.548), IL-6 (Fig. 6E; F(5,42) = 8.321, η^2^_p_ = 0.498), and TNF-α (Fig. 6F; F(5,42) = 8.045, η^2^_p_ = 0.489) levels in the NS group significantly increased when compared with those in the CON group (*p* < 0.004, *p* < 0.001, and *p* < 0.001, respectively). In all sample-administered groups, cytokines levels reduced relative to the NS group. Notably, IL-1β and IL-6 levels significantly decreased in the FLU (*p* < 0.002 and *p* < 0.001) and CRE/TAU groups (*p* < 0.007 and *p* < 0.002, respectively) compared to those in the NS group. In contrast, TNF-α levels significantly decreased in all sample groups (*p* < 0.001), except in the CRE group (*p* < 0.002), when compared to those in the NS group.

2.10 Effect of CRE and TAU on protein kinase B (Akt) and extracellular signal-regulated kinase (ERK)/cyclic AMP response element-binding protein (CREB)/brain-derived neurotrophic factor (BDNF) pathway in CMS-induced depressive mice.

**Figure 7:**
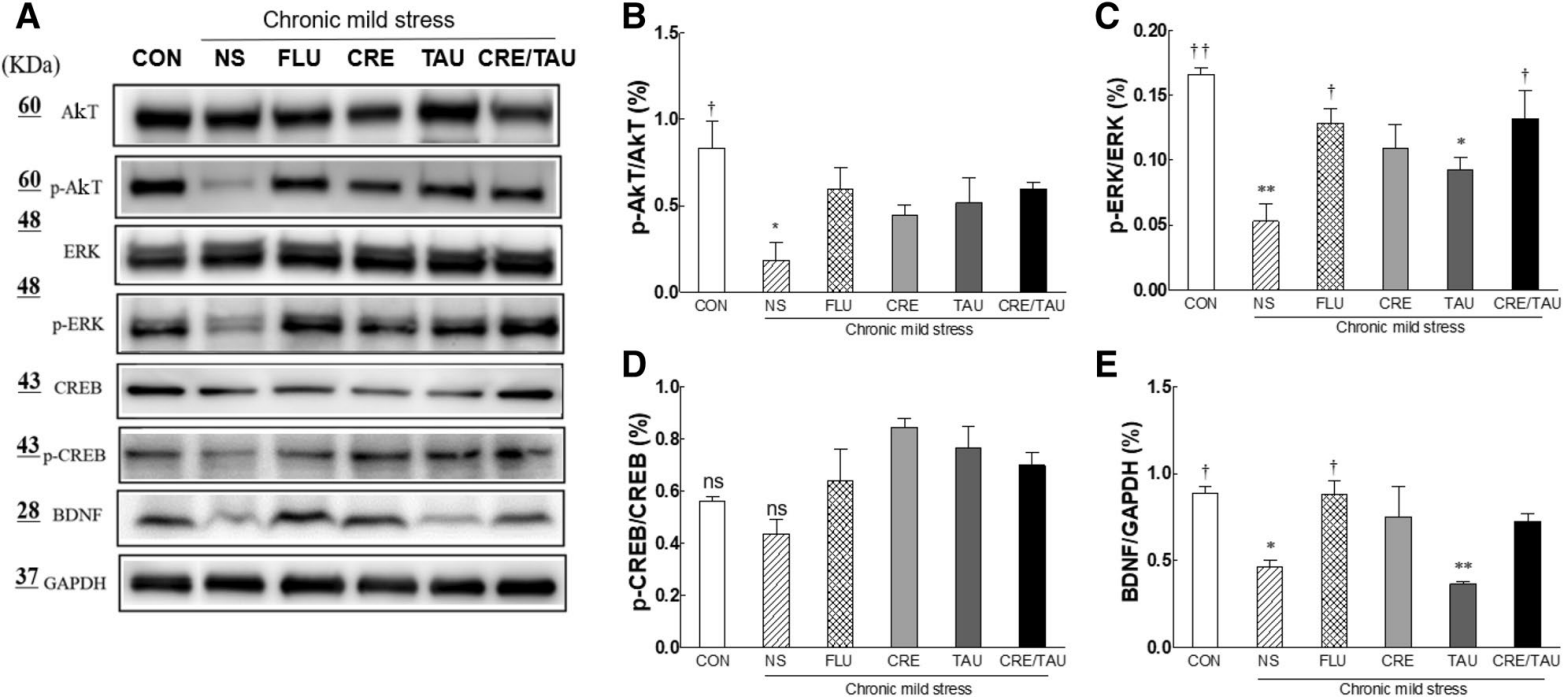
Effects of creatine, taurine, and the mixture of creatine and taurine on the expression of hippocampal signalling proteins in chronic mild stressed mice. (**A**) Western blot analysis shows the protein level of Akt, ERK, CREB, and BDNF. Represent expression level of (**B**) p-Akt/Akt, (**C**) p-ERK/ERK, (**D**) p-CREB/CREB, and (**E**) BDNF/GAPDH signalling proteins in chronic mild stressed mice. CON: non-stressed group (control), NS: stressed group (normal stress), FLU: 10 mg/kg of fluoxetine treatment (positive control), CRE: 7.5 mg/kg of creatine, TAU: 2.5 mg/kg of taurine, CRE/TAU: 7.5 mg/kg of creatine and 2.5 mg/kg of taurine mixture. Results are presented as the mean ± standard error of the mean (SEM) for each group (n = 8). *p < 0.05 and **p < 0.01 versus control group; ^†^*p* < 0.05 and ^††^*p* < 0.01 versus normal stress group (ANOVA followed by post-hoc Tukey’s test).

Western blotting was performed on the mouse hippocampal tissue to confirm the involvement of Akt and ERK/CREB/BDNF pathway in the mechanism of action of CRE and TAU. (Fig. 7A). Compared to the CON group, in the NS group, the expression level of the ratio of phosphorylated Akt (p-Akt) to intact total Akt significantly decreased (Fig. 7B; F(5,42) = 4.186, η^2^_p_ = 0.777, *p* < 0.04). The p-Akt/Akt expression ratio was higher in the NS group than in all sample-treated groups, with no significant difference. Similarly, the ratio of phosphorylated ERK (p-ERK)/ERK expression significantly decreased in the NS group when compared to the CON group (Fig. 7C; F(5,42) = 10.293, η^2^_p_ = 0.896, *p* < 0.005), and significantly increased in the CRE/TAU group compared to that in the NS group (*p* < 0.026) to a level similar to that observed in the FLU group. However, there were no significant differences between the phosphorylated CREB (p-CREB)/CREB protein expression levels in the experimental groups (Fig. 7D; F(5,42) = 1.075, η^2^_p_ = 0.473). Compared with the CON group, in the NS group, the protein expression level of BDNF significantly decreased (Fig. 7E, F(5,42) = 13.421, η^2^_p_ = 0.918, *p* < 0.018). BDNF protein expression levels significantly increased in the FLU group when compared with the NS group (*p* < 0.019). Additionally, the CRE and CRE/TAU groups showed an increase in the BDNF protein expression levels when compared to those in the NS group, but there was no significant difference.

## Discussion

The guanidine compound, CRE (*N*-aminoiminomethyl-*N*-methylglycine), is synthesised from glycine, arginine, and *S*-adenosylmethionine in the kidney, liver, pancreas, and brain. However, more than 50% of the CRE present in the body is dependent on dietary intake. The role of CRE in the muscular system is well known, but there is increasing evidence that it also has neuroprotective effect with potential to treat or alleviate central nervous system diseases, including depression^15,16^. CRE is a substrate for cytoplasmic and mitochondrial CRE kinases and plays a pivotal role in brain energy homeostasis^17^. Furthermore, there is an inverse correlation between the severity of depression and white matter CRE concentration in patients with depression and bipolar disorder^18^. According to a study indicating that mitochondrial brain abnormalities occur in depressed patients, CRE may be particularly suitable for mental illnesses^19,20^. Additionally, oral ingestion of CRE increases brain CRE levels by passing through the blood–brain barrier^21,22^, which may help predict the potential benefits of CRE supplementation in human patients with nervous system disorders.

Meanwhile, TAU is not a structural component of mammalian proteins but plays various physiological roles, including as an antioxidant, osmoregulator, membrane stabiliser, and neurotransmitter. Additionally, TAU demonstrates a neuroprotective effect against toxicities induced by excitatory amino acids^23^, and TAU levels in the cerebral cortex are often increased by forced swimming, in an animal model of depression^24^. The increase in TAU levels observed in these depression models could play an effective role in the brain. Conversely, studies have shown that high levels of TAU reduce behaviour^25^. Further, the OFTs confirmed locomotor activity of mice that received acute injections or chronic supplementation of TAU. The acute injection of TAU inhibited the motor activity of mice, while chronic supplementation increased motility^26^, which was caused by increased brain excitability through the changes in the inhibitory GABAergic system^27^. Therefore, we aimed to determine the appropriate ratio of TAU to the synergistic effect of CRE demonstrating an antidepressant effect.

In this study, we used a *Drosophila* model with depression owing to vibrational stress and selected the optimal ratio of CRE and TAU for antidepressant activity. Previous studies have shown that 3 d vibrational stress in *Drosophila* reduces spontaneous behavioural activity and induces a depression-like model^28^. Therefore, in the depression-like state of the vibration-induced *Drosophila* model, behavioural changes, locomotor activity, climbing activity, and survival rate were measured in the CRE, TAU, and CRE/TAU mixture groups. As a result, the performance of *Drosophila* that had reduced owing to vibrational stress increased in the CRE and TAU groups, especially in the 3:1 ratio CRE/TAU group (Figs. 1, 2, and 3).

Moreover, based on previous studies that induced depression from CMS in mice^29–31^, we confirmed the depressive effects and mechanism of action of the CRE and TAU mixtures. According to supplementary Tables 2 and 3, mice with CMS demonstrated a decrease in the body weight, while liver and hippocampal weights were restored to a significant level following CRE/TAU administration. Similarly, a study has shown that following CMS, the stress group mice demonstrated reduced body weight, and the weight of the fluoxetine-treated group (positive control) returned to normal levels^29^.

In the depressed animal model, the sucrose preference test, FST, OFT, and TST were representative for the behavioural analysis index for evaluating the antidepressant activity^32,33^. The sucrose preference test is a measure of anhedonia. Normal mice generally prefer an aqueous solution of sucrose over water; however, this preference decreases in rats exposed to long-term stress^34–36^. Additionally, exposure to stress significantly increased immobility in the FST when compared to the control group^29^, and stress groups demonstrated a decreased time in the central area of the OFT^30^. Similarly, in TST, chronic stress causes an increase in the number and immobility time^37^. Consistent with the results of previous studies, we identified reduced behaviour in CMS-induced depressive mice (Figs. 4, 5) and confirmed behavioural changes owing to CRE or TAU intake^30,37,38^. Additionally, a combination of CRE and TAU significantly alleviated the behavioural changes induced by depression.

Furthermore, stress and depression indicators include the level of monoamine, stress hormone, inflammatory factors, and nerve growth factors^39,40^. The monoamine hypothesis began with the discovery that approximately 15% of hypertensive patients prescribed reserpine demonstrated serious depression in the 1950s. In later studies, reserpine has been shown to reduce the monoamine content at brain synapses, leading to depression^41^; especially, symptoms manifested due to the depletion of serotonin and norepinephrine at the synapses^42^. In a previous study, monoamine levels in the cortex and hippocampus were measured using HPLC-electrochemical detection after acute stress and CMS. Here, norepinephrine, dopamine, and 5-HT levels were significantly lower in the unpredictable chronic stress group than in the control group^43^. Meanwhile, the TAU-supplemented group demonstrated an increase in the monoamine content to control levels^30^. Additionally, the results of this study confirmed that in the CRE/TAU mixture-treated group, the reduced monoamine levels owing to CMS were restored to a level similar to that in the CON group; this effect was not observed with CRE or TAU alone (Table 1).

Previous studies have shown a relationship between the stress paradigm and activation of the hypothalamus–pituitary–adrenal cortex axis. When people are in a stressful situation, the hypothalamus secretes corticotropin-releasing factor into the pituitary gland, which results in the pituitary gland secreting ACTH, which, in turn, secretes cortisol, the glucocorticoid hormone^44^. Several preclinical studies have suggested that activation of the immune system and depression may be bi-directional. Certain cytokines induce depression-like symptoms in rodents and primates^45,46^, and long-term stress changes the function of the immune system^47^ In this study, we observed that the expression of serum ACTH, corticosterone, CRH, and cytokines increased in CMS-induced depressive mice (Fig. 6). On the contrary, CRE/TAU mixtures effectively suppress the stress and immune response activated from depression.

Western blotting using hippocampal animal tissue confirmed the mechanism of the antidepressant activity of the test mixture. The protein expression levels of BDNF, Akt, p-Akt, ERK, p-ERK, CREB, and p-CREB were confirmed. Recently, several studies have shown changes in the intracellular signalling pathways following long-term antidepressant treatment^48–50^. Most antidepressants activate CREB through the activation of Akt and ERK, and ultimately increase BDNF expression and alleviate depression^51^. BDNF belongs to the family of nerve growth factors and is involved in the development of the nervous system. BDNF plays an important role in neural cell survival and differentiation, synapse formation, and cell function. Reportedly, the relationship between the BDNF concentration in the brain and the antidepressant effect has been reported^48^. Akt is closely related to neuronal differentiation and activated Akt protects the cell from necrosis and activates CREB^52^. Studies have shown that the expression and activity of ERK decrease in the brain of patients with depressive disorders and that the fluoxetine treatment reactivates ERK^53^. In this study, we observed that the expression levels of activated Akt, ERK, and BDNF were higher in the CRE/TAU-treated group than in the CRE or TAU groups (Fig. 7). The results of this study show a similar trend with previous studies regarding the effect of TAU on several signalling cascades in the hippocampus. Compared with normal rats, in TAU-treated rats, the ratio of protein expression of p-ERK1/ERK1, p-ERK2/ERK2, p-Akt/Akt, and p-CREB/CREB increased in the hippocampus^38^. Reportedly, the reduced BDNF levels induced by chronic corticosterone administration increased following the administration of CRE^54^.

This study demonstrated the antidepressant effect of CRE/TAU in *Drosophila* and rodent models. CRE/TAU effectively increased behaviour in the stress-induced depressive model, especially in animal models, with increased monoamine content and downregulation of stress hormones and inflammatory cytokines. This antidepressant effect of CRE/TAU can mediate the regulation of signalling proteins through the Akt and ERK/BDNF pathways. Treatment with CRE or TAU alone showed a similar effect to that using CRE/TAU mixture; however, the CRE/TAU 3:1 treatment group exhibited optimal antidepressant effects at low doses. To the best of our knowledge, this is the first study to demonstrate the antidepressant effects of an optimal ratio of CRE/TAU.

## Methods

### Materials

CRE and TAU were purchased from Sigma-Aldrich (St. Louis, MO); fluoxetine hydrochloride was purchased from Fluka (St. Louis, MO). All other chemicals and instruments were purchased from Sigma-Aldrich.

### *D. melanogaster* stocks

*D. melanogaster* Canton-S strain flies were obtained from the Bloomington Stock Center (Indiana University, Bloomington, IN, USA) and maintained in a 12/12 h light–dark cycle on the standard medium (water, cornmeal, sucrose, dried yeast, agar, *p*-hydroxybenzoic acid methyl ester, and propionic acid) in incubators (25 °C, 60% relative humidity). Male fly (2–5-day-old) was collected under CO_2_ anaesthesia for proper analysis. To assess locomotor activity and lifespan of CRE (ranging 0.05–0.20%), TAU (ranging 0.05–0.40%), and CRE/TAU mixtures (0.05/0.20%, 0.10/0.10%, and 0.15/0.05%), each concentration was added to the sucrose–agar medium (5% sucrose and 2% agar) and exposed for 3–6 d.

### Vibration stress procedure in *D. melanogaster*

Vibration stress treatment was partially modified based on the previous experimental model^22^. Flies were maintained in individual glass tubes (length, 60 mm; width, 3 mm) and placed in the vibration device (Brὓel & Kjoer, type 4,810) or incubator (control group). Vibrations (300 Hz) were applied for 20 s followed by a pause of 10 s and then repeated for 15 min, followed by a 30 min recovery time. The entire cycle was applied 12 times a day for 9 h, and stress was induced through vibrations for a total of 3 d during the week.

### Behavioural tests in *D. melanogaster*

To assess the antidepressant effect of CRE, TAU, and the mixture on the total activity of *Drosophila* activity monitoring (DAM) system (TriKinetics, Waltham, MA, USA) was used. DAM system analysed behavioural patterns over several days in individual flies. *Drosophila* was adapted in the tubes for 3 d under constant darkness. After adaptation, vibration stress was induced for 1 d and behavioural patterns were recorded for the next 1 d. Data were analysed using Actogram J Software, and sleep parameters were calculated by adding the number of total activities recorded daily^55^.

For video tracking and climbing activity analysis, adult male flies were subjected to vibration stress with a diet containing samples for 3 consecutive days. Two hours before video tracking, flies were individually placed in a circular arena (diameter, 8 mm; height, 0.1 mm) and stabilised in the dark phase. After adaptation, the flies in the arena were placed directly under a mounted digital video camera with a lamp board, followed by video recording for 5 min. The movement analysis of treated flies was performed using the Noldus EthoVision-XT system (Noldus Information Technology, Netherlands).

To measure climbing activity, the flies were transferred to an empty fly vial (25 × 100 mm) after vibration stress. The fly vials were divided into 8 distance zones (1–8 cm from the bottom to the top), and gently tapped at the bottom, stimulating flies to migrate from the bottom to each zone of the vials. The number of flies climbing up from the bottom was counted 1 min after tapping. The climbing ability assay was performed during the subjective night-time and daytime. Each test was repeated 10 times and continued after 10 min intervals^56^.

### Survival rate of *D. melanogaster*

To assess the survival rate, collected adult male flies were transferred to a vial containing the sample medium. Survivorship of flies was recorded every 2 d for 6 d; recorded data were further analysed and presented as the survival rate.

### Animals and housing conditions

Male BALB/c mice (4 weeks old, 18–22 g) were acquired from Orient Bio Inc. (Seongnam, Korea). Animals were housed in cages at 24 ± 2 °C and 55% relative humidity in a 12 h light/dark cycle and provided standard diet and water ad libitum. Animal use was conducted in accordance with the National Institute of Health Guide for the Care and Use of Laboratory Animals and approved by the Korea University Animal Care Committee (KUIACUC-2018–20, Seoul, Korea). Mice were divided into six experimental groups (CON, NS, fluoxetine, CRE, TAU, and CRE/TAU groups).

### CMS procedure in mice

The CMS method was partially modified based on a previously described experimental model^57,58^. Experimental animal stressors included water and food deprivation, an empty water bottle presentation, reversal of daytime and night-time cycles, crowded housing, tilted box (45°), bedding removal, and wet bedding. Experimental animals could not predict when and what kind of stress would be presented (Supplementary Table 1). Stressors were randomly scheduled over the 5-week experiment. For the first 2 weeks, only CMS was applied to the experimental animals. Then, for the next 3 weeks, mice were orally administered fluoxetine (10 mg/kg), CRE (7.5 mg/kg), TAU (2.5 mg/kg), or CRE/TAU [7.5/2.5 mg/kg (3:1 ratio)] with CMS application. The control group was raised in other home cages with freely supplied water and food, avoiding contact with the stressed group.

### Behavioural tests in mice

Behavioural analysis was performed using the sucrose preference, forced swim, open field, and tail suspension tests. Except for the sucrose preference test, the analysis was performed after 5 weeks of CMS exposure and sample treatment. The sucrose preference test was performed at two time points: after 2 weeks of CMS exposure and 5 weeks of CMS exposure and sample treatment.

The sucrose preference test was performed as described by Zhu et al.^58^ with modifications. Three days before the test, the mice were trained to adapt by providing a 1% sucrose solution (w/v) for 24 h. After providing tap water for 24 h, water and food were deprived for 24 h before testing. Mice housed in individual cages were given free access to two bottles containing 1% sucrose solution or water on the day of testing. After 24 h, the weight of sucrose solution and tap water consumed was recorded and sucrose preference was calculated using the following equation:

$${\text{Sucrose preference}}\,(\%)=1\%\,{\text{sucrose solution intake}}/({\text{water intake}} + 1\%\,{\text{sucrose solution intake}})\times 100.$$

The forced swimming test (FST) method was partially modified based on a previous experimental model^53^. Mice were forced to swim in a transparent acrylic cylinder (25 cm high, 10 cm diameter, and filled with 20 cm water at 23 ± 2 °C). The animals were adapted in the water for the first 2 min, and the animal behaviour was recorded using a video camera for 5 min. Immobilisation time of mice was measured using the Noldus EthoVision-XT system. Immobilisation state was defined by a minimum amount of head movement or floating without moving.

Locomotor activity was assessed using an open field test (OFT); after 3 min of adaptation to a black acrylic cage (25 × 25 × 30 cm), the activity of the mice was recorded for 10 min. Frequency and cumulative duration in the central compartment (15 × 15 cm), mobility, and horizontal ambulation were measured using the Noldus EthoVision-XT system.

The tail suspension test (TST) was partially modified based on a previous experimental model^59^. Experimental mice were individually suspended using their tails by tape to the top of the black box (30 × 30 × 50 cm) positioned horizontally 50 cm above the tabletop. Mice were adapted to the upside-down posture for 2 min. After adaptation, immobilisation time was measured for 5 min using the Noldus EthoVision-XT system. Immobilisation state implies that animal has completely stopped movement in the upside-down suspended state.

### Enzyme-linked immunosorbent assay (ELISA)

Following the TST, mice were rapidly euthanized, and 1 mL of blood was collected. The collected blood was centrifuged at 15,000 rpm at 4 °C for 15 min, and the supernatant was separated and stored at − 80 °C until analysis. Corticosterone levels were measured according to the manufacturer’s instructions using the Corticosterone ELISA kit (LDN, USA). ACTH level was measured by POMC ELISA kit (Aviva Systems Biology, USA), IL-1β levels using the IL-1β/IL-1F2 ELISA kit (R&D SYSTEMS, USA), IL-6 level by the IL-6 ELISA kit (BD Biosciences, USA), and TNF-α levels by the TNF ELISA kit (BD biosciences).

### HPLC analysis

For catecholamine and serotonin analysis, mice were euthanized, and the brains were rapidly removed, frozen in liquid nitrogen, and maintained at − 80 °C until assay. The brain was homogenised in 200 μL of 0.2 M perchloric acid containing 3 mM cysteine. The homogenate was centrifuged (12,000 × *g*, 10 min, 4 °C), and the resulting supernatant was left on ice for 5 min and analysed by HPLC-fluorescence detection (Waters Alliance 2,695 separations module, Milford, MA, USA) as previously described^60^. Catecholamine and serotonin levels were measured using the YMC-Pack Pro C18 column (250 × 4.6 mm, 5 μm) with acetate buffer (pH 3.5, 12 mM acetic acid, 0.26 mM Na_2_EDTA)–methanol (86:14, v/v) as the mobile phase. The flow rate was 1.0 mL/min. Catecholamine and serotonin detection was achieved using native fluorescence with excitation at 279 nm and emission at 320 nm.

### Quantitative reverse-transcription PCR (qRT-PCR)

For the CRH mRNA expression assay, the brain was obtained after euthanizing the experimental animals. Total RNA was extracted from mouse brains using TRIzol (Invitrogen, CA, USA), while genomic DNA was extracted using Direct-zol RNA Miniprep (ZYMO Research, CA, USA) according to the manufacturer’s protocol. Quality-controlled RNA (1 μg) was reverse transcribed using Super-Script III Reverse Transcriptase (Invitrogen) with oligo d(T) as the primer. The generated cDNA was subjected to qRT-PCR using a Power TaqMan PCR Master Mix kit (Applied Biosystems, CA, USA). The cycling conditions were 50 °C for 2 min, 95 °C for 10 min, followed by 40 cycles of 95 °C for 15 s, and 60 °C for 1 min. Quantitative analysis was conducted using the Step-One-Plus Software version 2.0 (Applied Biosystems)^61^. The endogenous housekeeping gene, GAPDH, was used for result normalisation. The following primer sequences were used for qRT-PCR: mouse GAPDH (NM_008084.2), 5′-CATGGCCTTCCGTGTTCCTA-3′ (forward) and 5′-GCGGCACGTCAGAT CCA-3′ (reverse); and mouse CRH (NM_205769.2), 5′-ACCAAGGGAGGAGAAGAGAGCG-3′ (forward) and 5′-GCTGCTCCGGCTGCAAGAAA-3′ (reverse).

### Western blot analysis

For western blotting, all experimental animals were euthanized, and the hippocampus was isolated to separate the proteins. Isolated protein concentrations were determined using the BCA assay. Then, 50 μg of protein was electrophoresed on 4–15% Tris–glycine polyacrylamide gels (Bio-rad Laboratories, Inc., Hercules, CA, USA), transferred to Immobilon-P (Millipore Corporation, Bedfrom, MA) polyvinylidene difluoride membranes, blocked for 1 h in 5% bovine serum albumin, and incubated overnight at 4 °C with either anti-Akt (Cell Signaling, #9272, 1:1000), anti-p-Akt (Cell Signaling, #9271, 1:1000), anti-ERK1/2 (Invitrogen, 13-6200, 1:1000), anti-pERK1/2 (Cell Signaling, #9101, 1:1000), anti-CREB (Cell Signaling, #9197, 1:1000), anti-pCREB (Cell Signaling, #9,191, 1:1,000), anti-BDNF (Invitrogen, 710,306, 1:1,000), or GAPDH (Cell Signaling, #5174, 1:1000). Membranes were washed 3 times with Tris-buffered saline with 0.1% Tween 20 (TBST) and incubated for 1 h at 25 °C with horseradish peroxide-conjugated IgG secondary antibody (Cell Signaling, #5157, 1:2000). Signals were developed using ECL Prime (GE Healthcare Life Sciences, CT, USA) on a FluorChem E Imaging System (Protein Simple, California USA). Gel documentation and relative quantification were performed with Image J software. The quantity of proteins loaded on gel was controlled using the housekeeping protein GAPDH^62–64^.

### Data analysis

Experimental results were expressed as mean ± standard error of the mean. Statistically significant differences among the groups were determined by one-way analysis of variance, followed by post hoc Tukey's multiple range test at the 5% significance level, using the Statistical Package for Social Sciences version 12.0 (SPSS Inc., Chicago, IL, USA). Each *p* value with partial eta-squared estimates of effect size are given in the Result Section.

## Supplementary information

Supplementary information.
