# Supplementary material for: Creatine and taurine mixtures alleviate depressive-like behaviour in Drosophila melanogaster and mice via regulating Akt and ERK/BDNF pathways
Source: Sci Rep. 2020 Jul 9;10:11370. doi: 10.1038/s41598-020-68424-1 (PMC7347602; doi:10.1038/s41598-020-68424-1)
Supplement: Supplementary file 1 — Supplementary information. [file 41598_2020_68424_MOESM1_ESM.pdf]

Creatine and taurine mixtures alleviate depressive-like behaviour in *Drosophila melanogaster* and mice via regulating AKT and ERK/BDNF pathways

Suhyeon Kim[1], **Ki-Bae Hong**[2], **Singeun Kim**[1], **Hyung Joo Suh**[1,2,*] **and Kyungae Jo**[2,*]

[1]Department of Public Health Science, Korea University, Seoul 02841, Republic of Korea

[2]BK21Plus, College of Health Science, Korea University, Seoul 02841, Republic of Korea

Supplementary Table 1. Chronic mild stress (CMS) procedure

Week	MON	TUE	WED	THU	FRI	SAT	SUN
1	SC (9 h)<br>OI (12 h)	CH (24 h)<br>CT (16 h)	WC (24 h)	SC (24 h)<br>FST (5 min)	CT (24 h)<br>CH (24 h)	WC (9 h)	WFD (24 h)
2	CT (24 h)<br>RAF (3 h)<br>OI (12 h)	CH (24 h)<br>CT (16 h)	WC (24 h)<br>WD (16 h)	CH (24 h)<br>FD (16 h)	SC (24 h)<br>FD (16 h)	WC (9 h)	WFD (24 h)
3	SC (24 h)<br>EWB (3 h)<br>OI (12 h)	WC (24 h)<br>FD (16 h)	SC (24 h)<br>OI (12 h)<br>RAF (3 h)	CH (24 h)<br>CT (9 h)	CT (24 h)<br>FST (5 min)	WC (9 h)	WFD (24 h)
4	CT (24 h)<br>RAF (3 h)<br>OI (12 h)	WC (24 h)<br>WD (16 h)	CH (24 h)<br>CT (16 h)	SC (24 h)<br>FST (5 min)	CT (24 h)<br>CH (24 h)	WC (9 h)	WFD (24 h)
5	SC (24 h)<br>EWB (3 h)<br>OI (12 h)	CH (24 h)<br>CT (16 h)	WC (24 h)<br>FD (16 h)	SC (24 h)<br>OI (12 h)<br>RAF (3 h)	CT (24 h)<br>CH(24 h)	WC (9 h)	WFD (24 h)

WFD: water/food deprivation, WD: water deprivation, FD: food deprivation, EWB: empty water bottle, WC: wet cage, SC: solid cage, CT: cage tilt, CH: crowded housing, RAF: restricted access to food, OI: overnight illumination, FST: forced swimming

Supplementary Table 2. Effects of creatine, taurine, and the mixture of creatine and taurine on body weight of chronic mild stressed mice

Body weight (g)		Group					
		**NOR**	**CON**	**FLU**	**CRE**	**TAU**	**CRE/TAU**
Week	1st	22.93±0.64[a]	22.83±0.45[b]	22.55±0.52[b]	22.41±0.56[b]	22.40±0.49[b]	21.86±0.27[c]
	2nd	25.33±0.42[a]	23.84±0.46[c]	24.19±0.42[b]	23.89±0.51[bc]	24.14±0.35[b]	24.11±0.38[b]
	3rd	25.91±0.40[a]	25.00±0.61[b]	25.19±0.39[b]	24.75±0.41[c]	24.81±0.46[c]	24.79±0.47[c]
	4th	26.46±0.40[a]	24.97±0.60[c]	25.48±0.39[b]	24.54±0.28[b]	24.75±0.38[c]	25.26±0.41[b]
	5th	26.90±0.34[a]	23.11±0.53[c]	25.81±0.59[b]	25.06±0.45[bc]	24.89±0.33[bc]	25.65±0.31[b]

NOR: non-stressed group (normal), CON: stressed group (control), FLU: 10 mg/kg of fluoxetine treatment (positive control), CRE: 7.5 mg/kg of creatine, TAU: 2.5 mg/kg of taurine, CRE/TAU: 7.5 mg/kg of creatine and 2.5 mg/kg of taurine mixture. Results are presented as the mean ± standard error of the mean (SEM) for each group (n=8). Different letters indicate significant differences ($p<0.05$) among samples by one-way ANOVA followed by post-hoc Tukey's test.

Supplementary Table 3. Effects of creatine, taurine, and the mixture of creatine and taurine on organ weight of chronic mild stressed mice

Organ weight (g/100 g of body weight)	Group					
	**NOR**	**CON**	**FLU**	**CRE**	**TAU**	**CRE/TAU**
Kidney	1.56±0.01^{ns}	1.58±0.01	1.61±0.01	1.59±0.01	1.63±0.01	1.61±0.01
Spleen	0.28±0.01^{ns}	0.27±0.01	0.26±0.01	0.28±0.01	0.28±0.02	0.27±0.01
Liver	5.02±0.03^{a}	4.64±0.04^{b}	5.16±0.04^{ab}	5.20±0.04^{ab}	5.32±0.04^{a}	5.18±0.03^{ab}
Heart	0.65±0.01^{ns}	0.65±0.01	0.63±0.01	0.64±0.01	0.65±0.02	0.64±0.01
Total brain	1.52±0.03^{ns}	1.67±0.06	1.61±0.07	1.66±0.02	1.64±0.05	1.62±0.06
Hippocampus	0.13±0.21^{a}	0.11±0.12^{b}	0.13±0.16^{a}	0.12±0.14^{ab}	0.13±0.17^{a}	0.13±0.16^{a}

NOR: non-stressed group (normal), CON: stressed group (control), FLU: 10 mg/kg of fluoxetine treatment (positive control), CRE: 7.5 mg/kg of creatine, TAU: 2.5 mg/kg of taurine, CRE/TAU: 7.5 mg/kg of creatine and 2.5 mg/kg of taurine mixture. Results are presented as the mean ± standard error of the mean (SEM) for each group (n=8). Different letters indicate significant differences ($p<0.05$) among samples by one-way ANOVA followed by post-hoc Tukey's test.

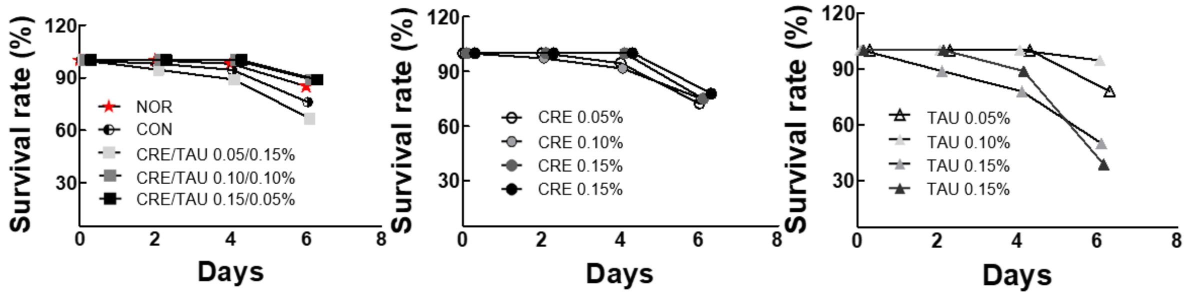

Supplementary Figure 1. Effects of creatine, taurine, and the mixture of creatine and taurine on survival rate in vibration stressed Drosophila melanogaster. Experiments analysed the survival rate during 6 days in the non-stressed group, stressed group, creatine-treated groups (0.05, 0.10, 0.15, and 0.20%), taurine-treated groups (0.05, 0.10, 0.20, and 0.40%), mixture of creatine and taurine-treated groups (0.05/0.15, 0.10/0.10, and 0.15/0.05%) with vibration stress. Results are presented as the mean ± standard error of the mean (SEM) for each group. Different letters indicate significant differences ($p<0.05$) among samples by one-way ANOVA followed by post-hoc Tukey’s test.
